# Diagnostic Challenges of Cyclosporiasis in Chronic Diarrhea: A Case Study

**DOI:** 10.3390/microorganisms13092209

**Published:** 2025-09-21

**Authors:** Estera Banasik, Agnieszka Dobrowolska, Lucyna Woźnicka-Leśkiewicz, Piotr Eder

**Affiliations:** 1 Department of Gastroenterology, Dietetics and Internal Medicine, Poznan University of Medical Sciences, University Clinical Hospital, 60-355 Poznan, Poland; agdob@ump.edu.pl (A.D.); piotreder@ump.edu.pl (P.E.); 2 Department of Family Medicine, Poznan University of Medical Sciences, 60-355 Poznan, Poland; lucyna.woznicka@gmail.com

**Keywords:** *Cyclospora cayetanensis*, ulcerative colitis, vedolizumab, opportunistic infection

## Abstract

Cyclosporiasis, caused by *Cyclospora cayetanensis*, is a rare opportunistic infection, particularly in immunosuppressed patients with inflammatory bowel disease (IBD). Its clinical presentation may mimic IBD, with chronic diarrhea and anemia resistant to standard therapy. We report the case of a 24-year-old woman with ulcerative colitis (UC) and a history of liver transplantation, treated with vedolizumab and immunosuppressants. Despite endoscopic remission, she experienced persistent abdominal pain, diarrhea, and iron deficiency anemia. Escalation of biologic therapy was ineffective. After exclusion of bacterial and viral causes, stool testing identified *Cyclospora cayetanensis*. Treatment with nitazoxanide led to rapid clinical and laboratory improvement. Biologic therapy was temporarily discontinued and later resumed without recurrence of symptoms. This case shows that chronic diarrhea in IBD patients may not always result from the underlying disease. Immunosuppression increases the risk of opportunistic infections. Early diagnosis and specific treatment can improve outcomes and allow safe continuation of IBD therapy.

## 1. Introduction

Cyclosporiasis is a parasitic disease caused by *Cyclospora cayetanensis*, an intestinal protozoan from the coccidia group. The parasite replicates inside enterocytes of the small intestine, and oocysts are excreted in feces. They become infectious only after several days of sporulation in the environment, which requires warm and humid conditions. Transmission occurs via the fecal–oral route, most often through contaminated food or water [1,2,3,4,5,6,7]. The disease is endemic in tropical and subtropical regions of Asia, Africa, and Latin America, while in Europe and North America, most cases are linked to travel or imported produce [1,2,3,4,5,6,7].

*Cyclospora* infection can damage the intestinal barrier and cause inflammation, malabsorption, and symptoms such as chronic diarrhea, abdominal pain, nausea, and fatigue [1,2,3,4,5,6]. In immunocompetent individuals, the infection is usually self-limiting, but in immunocompromised patients, it may be severe and prolonged [1,2,3,4,5,6]. Patients with autoimmune diseases or after organ transplantation who receive immunosuppressive therapy are at particular risk of this opportunistic infection and may present with an atypical course [1,2,3,4,5,6,7,8,9,10,11,12,13,14,15,16,17,18,19]. In such cases, cyclosporiasis may mimic an inflammatory bowel disease (IBD) flare or malabsorption syndrome [8,9,10,11], which can delay correct diagnosis and lead to unnecessary escalation of IBD treatment [8,9,10,11,15,16,17,18,19].

Diagnosis is based on stool examination using specific techniques like Ziehl-Neelsen staining, and at least three samples collected at 48 h intervals [1,2,5]. In uncertain cases, molecular tests such as PCR (Polymerase Chain Reaction) can be used to detect *C. cayetanensis* DNA in stool samples. These methods are more sensitive and specific than microscopy but are usually reserved for patients with negative stool tests and strong clinical suspicion [3,12,13,20]. Treatment is generally effective. The first-line therapy is trimethoprim-sulfamethoxazole. In cases of intolerance or resistance, alternative treatment includes nitazoxanide or ciprofloxacin [1,3,4]. Early treatment is important to prevent complications, especially in immunocompromised patients. No vaccine is currently available, so prevention depends on avoiding potentially contaminated food and water [1,3,4].

Opportunistic infections are increasingly recognized in patients with IBD, but parasitic infections remain underdiagnosed and underreported in this population [8,9,10,11,15,16]. Immunosuppression, mucosal barrier injury, and immune dysregulation increase susceptibility to these opportunistic pathogens [8,9,10,11,12,13,14,15,16]. According to the Second European Evidence-Based Consensus on the Prevention, Diagnosis and Management of Opportunistic Infections in IBD, such infections are caused by organisms that rarely cause disease in healthy hosts but may result in significant morbidity in immunocompromised patients [15,16].

Here we report a rare case of *Cyclospora cayetanensis* infection in a patient with ulcerative colitis (UC) treated with vedolizumab, who developed persistent diarrhea and anemia despite partial endoscopic response. Vedolizumab is a gut-selective humanized monoclonal antibody against α4β7 integrin and is associated with a lower risk of systemic infections compared to anti-Tumor Necrosis Factor (TNF) or Janus Kinase (JAK) inhibitors [21,22]. However, its localized immunosuppressive effect on the gastrointestinal tract may increase susceptibility to enteric infections, especially in the context of imported food consumption or travel to endemic areas [1,2,3,4,20]. Data on parasitic infections in vedolizumab-treated patients are scarce [21,22]. This case highlights the importance of considering parasitic infections in the differential diagnosis of persistent or atypical symptoms in patients with IBD [1,2,3,4,8,9,10,11,15,16,20].

## 2. Case Description

A 24-year-old woman with a history of UC, diagnosed in April 2020, was admitted to the hospital due to a flare despite ongoing biological treatment with vedolizumab. Colonoscopy performed during the qualification for biologic therapy revealed pancolitis with extensive erosive lesions, small ulcerations, and contact bleeding (E3 according to the Montreal classification, Mayo endoscopic subscore 3) [23,24].

The diagnosis of UC was based on clinical symptoms (recurrent bloody diarrhea, abdominal pain, and severe iron-deficiency anemia), elevated stool calprotectin (>800 μg/g), CT evidence of colon wall thickening with loss of haustration, colonoscopy showing multiple small ulcers and mucosal contact bleeding, and histology showing crypt distortion, irregular crypt arrangement, mucosal atrophy, and inflammatory cell infiltrates with plasma cells and eosinophils.

The disease was classified as E3, S3 (Montreal classification) with Mayo subscore of 3 [23]. Four months earlier, a colonoscopy showed only mild UC changes (Mayo 1), but later, continuous colonic involvement and histology confirmed UC [23,24].

The initial disease course was unfavorable, with chronic diarrhea (up to eight loose stools per day with visible blood) and anemia unresponsive to oral supplementation. An iron absorption test conducted one year earlier showed normal results. Following an intravenous administration of ferric derisomaltose, the patient developed an anaphylactic reaction. Due to persistent anemia, she received six units of red blood cell (RBC) transfusions over five months prior to the current hospitalization.

The patient had autoimmune hepatitis in childhood, which led to liver transplantation in 2012 due to acute liver failure and retransplantation in 2018 because of right-lobe cirrhosis. Since the first transplantation, she has been on long-term post-transplant immunosuppression with tacrolimus (4 mg/day) and deflazacort (6 mg/day). Azathioprine had been used before the first transplant for autoimmune hepatitis and was reintroduced at a dose of 50 mg/day in May 2020 after the diagnosis of ulcerative colitis. Mycophenolate mofetil, previously part of the post-transplant regimen, was discontinued when azathioprine was restarted, as advised by the transplant clinic. Mesalazine was administered only briefly (1–2 months) but was stopped due to adverse effects (headaches and palpitations). At the time of *C. cayetanensis* infection, the patient had been on triple immunosuppressive therapy (tacrolimus, deflazacort, and azathioprine) for 7 months. Due to poor clinical response and advanced endoscopic lesions (Mayo score 3), the patient was started on vedolizumab therapy, considering her profound immunosuppression and the gut-selective mechanism of action of the drug [16,25].

Routine laboratory tests revealed elevated CRP 5.8 mg/L (ref. < 5 mg/L), leukocytosis 13.17 × 10^3^/μL (ref. 3.90–11.00/μL), microcytic anemia with hemoglobin 8.6 g/dL (ref. 12.0–15.6 g/dL; MCV 68.7 fL, ref. 80.00–99.00 fL), thrombocytosis 665 × 10^3^/μL (ref. 130–400 × 10^3^/μL), and mildly elevated creatinine 1.16 mg/dL (ref. 0.50–0.90 mg/dL). Iron and ferritin levels were within normal limits. Fecal calprotectin exceeded 800 μg/g (ref. lower than 50 μg/g) [14,16]. Contrast-enhanced CT of the abdomen and pelvis revealed rectal wall thickening, loss of haustration, and multiple enlarged mesenteric lymph nodes up to 10 mm (ref. lower than 5 mm). Initial infectious screening (HbsAg, total anti-Hbc, anti-HCV, p24 antigen/anti-HIV-1/2 antibodies, *Clostridioides difficile* toxins A and B, Quantiferon test) was negative, and vedolizumab was initiated [17,18].

Follow-up laboratory tests before the first and second vedolizumab doses showed a decreasing trend in hemoglobin (7.2 g/dL; 6.9 g/dL), while CRP declined (6.1 mg/L; 4.9 mg/L). Before the third dose, the patient was hospitalized again due to severe anemia and iron deficiency. She received two units of RBCs but refused intravenous iron due to a history of anaphylaxis. The patient was discharged with oral iron supplementation at 80 mg/day.

Before the fourth dose, anemia persisted (Hb: 8.2 g/dL) with monocytosis, eosinophilia, and increased CRP (18.2 mg/L).

Prior to the fifth dose, bowel movements had decreased to 3–5 loose stools per day, and abdominal pain had resolved, but hemoglobin dropped 6.8 g/dL, eosinophilia persisted, and platelet count rose to 521 × 10^3^/μL; CRP was within normal limits. The dosing interval was reduced to every 4 weeks, and anemia workup was expanded.

**Figure 1:**
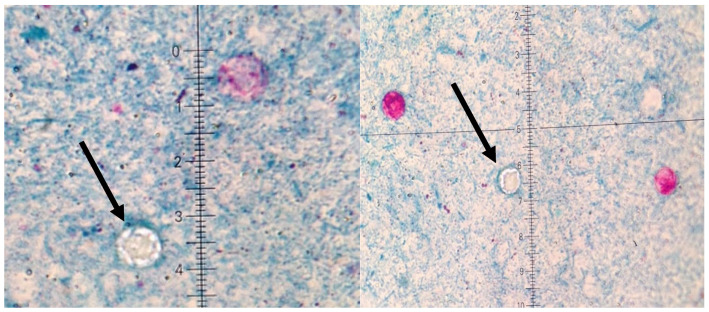
Microscopic preparations of stools stained using the Ziehl–Neelsen method. *Cyclospora cayetanesis* is indicated by arrows.

A significant clinical deterioration occurred after the fifth dose of vedolizumab, prompting the current hospitalization. Upon admission, the patient reported a two-week history of worsening diarrhea (up to 10 stools/day with mucus), central abdominal pain, loss of appetite, and fatigue. On physical examination, she appeared pale, dehydrated, BMI of 19.6 kg/m^2^ with hyperactive bowel sounds. Differential diagnosis was conducted with a wide range of laboratory, microbiological, and imaging tests being performed (Appendix A, Table A1). Laboratory results revealed: severe anemia (Hb 6.7 g/dL, MCV 68 fL), leukocytosis (14.37 × 10^3^/μL), elevated CRP (99.4 mg/L), acute kidney injury (creatinine 3.38 mg/dL, ref. 0.57–1.11 mg/dL; urea 104 mg/dL, ref. 17.00–50.00 mg/dL), and fecal calprotectin was elevated of 190 μg/g. Liver enzymes (AST, ALT), bilirubin, ALP, GGT, total protein, albumin, and tissue transglutaminase IgA antibodies were normal (IgA levels were within the reference range). Tacrolimus level was within therapeutic range. Stool tests for *Clostridioides difficile* toxin A and B were negative. Stool cultures for *Salmonella*, *Shigella*, enteropathogenic *Escherichia coli*, and *Yersinia enterocolitica* were negative. Three stool samples tested negative for amoebiasis and other parasites (Appendix A, Table A1). However, Ziehl-Neelsen staining revealed *Cyclospora cayetanensis* oocysts in all three samples (approximately two per 1000 × field) (Figure 1) [1,2,22].

Abdominal ultrasound revealed mesenteric lymphadenopathy to 6–7 mm (normal < 5 mm) and thickening of the colonic wall up to 5 mm (normal < 3 mm) in the cecum and ascending colon and transverse colon. A QuantiFERON test was negative. Due to persistent anemia, an upper endoscopy was performed, revealing a few gastric erosions; the urease test was negative. Duodenal biopsies were taken for malabsorption assessment, and histopathology showed increased intraepithelial lymphocytes (33/100 enterocytes), consistent with Marsh 0 (Table 1).

Because of ongoing disease activity and risk of perforation, only a rectosigmoidoscopy was performed, revealing loss of vascular pattern in the sigmoid colon (Mayo 1). Colonic biopsy showed chronic inflammation of mild intensity (previously severe) with scattered eosinophils and occasional branching crypts (Table 1).

The patient was transferred to the Department of Tropical and Parasitic Diseases, where nitazoxanide was initiated (500 mg orally, three times daily for 10 days), as selected by an infectious disease specialist. Immunosuppressive therapy with tacrolimus and azathioprine was continued. Significant clinical improvement was observed within three days, including a reduction in bowel movements and resolution of abdominal pain. Over the following weeks, inflammatory markers declined, renal function improved, and hemoglobin stabilized (Table 2).

After finishing treatment, no follow-up stool tests were performed to confirm eradication of *Cyclospora cayetanensis*. This decision was justified by the clear clinical improvement, including resolution of diarrhea and abdominal pain as well as normalization of laboratory results. As reported in the literature, oocysts may still be seen in stool for some time and do not always reflect active infection; therefore, if symptoms resolve, routine follow-up stool testing is not required [4,6].

After the diagnosis of cyclosporiasis, vedolizumab was withheld for 4 weeks, as biologic therapy is not recommended during active infection. The effect of vedolizumab in this setting is not well established, and the patient’s intestinal condition allowed for a temporary interruption. Approximately four weeks after completing antiparasitic therapy, vedolizumab was reintroduced due to the initially severe UC and the favorable endoscopic response that had been achieved before the infection. Following reintroduction, abdominal pain resolved, and stool frequency decreased to 6 per day (without blood), although CRP remained elevated (18.2 mg/L) and anemia persisted (Hb 7.8 g/dL, MCV normalized).

After eight weeks, bowel movements decreased further (4–5/day, without blood), the patient reported improved well-being, and CRP declined to 6.8 mg/L. Platelet count increased to 519 × 10^3^/μL.

Twelve weeks post-infection, she was doing well. Occasional abdominal pain persisted. Stools were 2–5/day, without blood. CPP normalized, and hemoglobin increased to 10.5 g/dL (MCV within normal limits). Mild anemia persisted but did not require transfusions (Table 2).

Six months after the antiparasitic treatment, the patient was in good general condition, with stable hemoglobin levels and mild gastrointestinal complaints.

## 3. Discussion

This case underscores several important clinical insights regarding the diagnosis and management of *Cyclospora cayetanensis* infection in patients with IBD. Opportunistic infections may mimic disease flares in patients with IBD, especially those receiving immunosuppressive therapy, leading to misinterpretation and unnecessary treatment escalation [8,9,10,11,17].

The patient’s symptoms initially resembled a moderate UC flare. However, stool microscopy using Ziehl-Neelsen stain revealed *Cyclospora* oocysts, which was crucial for establishing the correct diagnosis [1,4,5,6]. In the setting of biologic therapy, distinguishing infection from IBD exacerbation is essential [8,9,10,11,17,18]. In cyclosporiasis, the lack of distinct endoscopic features—such as deep ulcerations or pseudomembranes—can lead to misinterpretation of endoscopic findings [1,4,5,6]. Although vedolizumab, a gut-selective α4β7 integrin antagonist, is associated with a lower risk of systemic infections compared to anti-TNF agents [17,18,21,22], its localized impact on mucosal immunity may still predispose patients to enteric infections, particularly in ulcerative colitis. Thus, the overall risk of intestinal infections may not be significantly reduced [18]. Importantly, our patient had no recent travel history, suggesting local or imported foodborne exposure [2,4,7].

It is also possible that the gastrointestinal symptoms were at least partly related to long-term immunosuppressive therapy after liver transplantation. Chronic immunosuppression can alter the gut microbiota, increase intestinal permeability, and weaken local immune defense, which may lead to dysbiosis. Through the gut–liver axis, these changes may also impair hepatic immune responses and influence the course of IBD. In our case, both intestinal injury and impaired hepatic immune could have increased susceptibility to opportunistic intestinal infections and exacerbated colonic inflammation. Since dysbiosis is increasingly recognized as an important factor in IBD, microbiota-targeted therapies, including fecal microbiota transplantation (FMT), may represent a potential option in complex cases, although current evidence in transplant recipients is still limited [27,28,29].

Another possible explanation is that *C. cayetanensis* infection was present in a subclinical form before vedolizumab was started [21,22]. Vedolizumab works by blocking the interaction between the α4β7 integrin on T cells and MAdCAM-1 (Mucosal Addressin Cell Adhesion Molecule-1) on intestinal endothelial cells, preventing lymphocytes from migrating into the gut [21,22]. This reduces the influx of inflammatory cells and improves colonic inflammation in ulcerative colitis, but at the same time weakens local mucosal immunity [17,18]. As a result, a previously latent infection may have become clinically manifest after initiation of therapy [21].

Although cyclosporiasis is usually self-limiting in immunocompetent individuals, it can be protracted in immunosuppressed patients, resulting in significant morbidity. In our case, prompt recognition and targeted treatment with nitazoxanide led to rapid clinical improvement [4,5]. This highlights the importance of early recognition and targeted antimicrobial therapy.

Platelet counts remained high (483–665 × 10^3^/μL), most likely as a reactive change to inflammation and iron-deficiency anemia, both typical in active UC. The infection may have played a role, but the increase preceded *Cyclospora*, indicating UC activity and anemia as the main causes.

In contrast to CMV colitis, another opportunistic infection in IBD patients, *Cyclospora* infection does not cause cytopathic changes on histology and often lacks endoscopic abnormalities [17,18]. Thus, diagnosis relies on high clinical suspicion and appropriate microbiological testing. In our case, stool microscopy using Ziehl–Neelsen stain was diagnostic. Molecular tests may be more sensitive but are not widely available in routine settings [3,6].

To the best of our knowledge, this is the first reported case of *Cyclospora* infection in a patient with UC treated with vedolizumab. Although histological confirmation is not routinely required for the diagnosis of cyclosporiasis [3,6], the resolution of symptoms after antiparasitic treatment supports a causative role [5,6]. This case underscores the need for thorough infectious workups before escalating therapy in patients with IBD and diarrhea [8,9,10,11,17,18].

Clinicians should maintain a broad differential diagnosis when managing persistent diarrhea in IBD patients, particularly in those on biologics or with a history of immunosuppression. Parasitic infections should be considered even in non-endemic regions and in patients without travel history. Greater awareness of such rare enteric infections in IBD is essential to avoid unnecessary treatment escalation and optimize patient outcomes [6,19,25].

## 4. Conclusions

Although rare, parasitic infections can be difficult to recognize in patients with inflammatory bowel disease, especially when symptoms resemble a disease flare. This case shows the importance of looking carefully at ongoing digestive symptoms in immunosuppressed patients, even if they have not traveled to tropical areas. Vedolizumab reduces intestinal inflammation but decreases mucosal immunity, which may unmask latent or subclinical enteric infections, such as *Cyclospora cayetanensis*. Our report underscores the need to raise awareness among clinicians in non-endemic regions, such as Europe the clinical presentation of parasitic diseases. Greater awareness of such infections and better access to diagnostic tests may help with earlier diagnosis and prevent unnecessary treatment changes. Sharing cases like this can support clinical decision-making and improve care for patients treated with biologic therapies.

## Figures and Tables

**Table 1:** Endoscopic and histopathological findings during *Cyclospora cayetanensis* infection.

Endoscopy	Images	Histopathology
Gastroscopy: Revealed a normal appearance of the upper gastrointestinal tract.	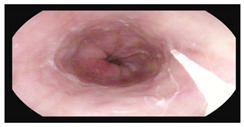 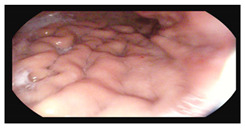 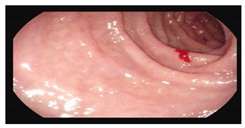	Fragments of duodenal mucosa with preserved architecture. The number of intraepithelial lymphocytes was estimated at 29–32 per 100 enterocytes, which exceeds the accepted upper limit of normal <25/100 enterocytes [26]. Lamina propria with numerous lymphocytes, plasma cells, and single eosinophils. Marsh type 0 classification.
Colonoscopy (Mayo subscore 1): Endoscopy revealed mucosal edema of the sigmoid colon with loss of the normal vascular pattern.	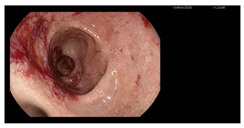 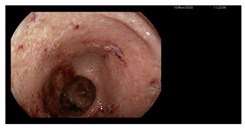 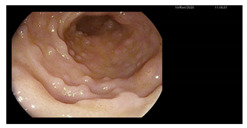	Mild architectural distortion of colonic crypts. Chronic inflammatory process of lamina propria with low-grade activity, presence of scattered eosinophils, and neutrophils dispersed in the lamina propria. No evidence of dysplasia, granulomas, or sarcoid-like lesions.

**Table 2:** Laboratory parameters during the clinical course.

Parameter/Time	UC Diagnosis	Before 1st Dose Vedolizumab	Before 2nd Dose	Before 4th Dose	Before 5th Dose	Cyclospora Infection	8 Weeks Post Infection	12 Weeks Post Infection
WBC (3.90–11.00) [×10^3^/μL)]	13.17	11.85	11.20	9.02	9.09	14.37	14.70	12.14
RBC (3.50–5.20) [×10^6^/μL]	4.31	3.32	3.12	3.53	3.11	3.31	3.42	3.62
Hgb (12.0–15.6) [g/dL]	8.6	7.2	6.9	8.2	6.8	6.7	9.4	10.5
MCV (80.0–99.0) [fL]	68.7	74.7	73.7	74.8	74.0	68.0	91.8	94.5
PLT (130–400) [×10^3^/μL)]	665	667	607	483	521	614	519	547
Fe (37–145) [μg/dL]	56	-	-	-	-	8	-	-
CRP (<5.0) [mg/L]	5.8	6.1	4.9	18.2	2.9	99.4	6.8	4.0
Creatynin (0.50–0.90) [mg/dL]	1.16	1.07	1.04	0.96	0.93	3.38	0.93	1.03
Calprotectin (<50) [μg/g]	>800.00	-	-	-	-	190.00	-	-

Abbreviations: WBC—white blood count; RBC—red blood cell count; Hgb—hemoglobin; MCV—mean corpuscular volume; PLT—platelets; Fe—serum iron; CRP—C-reactive protein; Calprotectin level—fecal calprotectin, UC—ulcerative colitis.

## Data Availability

The original contributions presented in this study are included in the article. Further inquiries can be directed to the corresponding author.

## Abbreviations

The following abbreviations are used in this manuscript:

IBD	Inflammatory Bowel Disease
UC	Ulcerative colitis
PCR	Polymerase chain reaction
DNA	Deoxyribonucleic acid
TNF	Tumor Necrosis Factor
JAK	Janus Kinase inhibitors
RBC	Red Blood Cell
CRP	C-reactive protein
MCV	Mean Corpuscular Volume
CT	Computed Tomography
BMI	Body Mass Index
Hb	Hemoglobin
CMV	Cytomegalovirus

## Appendix A

**Table A1:** All diagnostic procedures were performed according to international and institutional standards. The diagnostic workflow included blood tests, serological screening, stool examinations, endoscopy with biopsy, histopathology, and imaging.

Diagnostic Procedure	Details
Blood tests	Complete blood count (CBC) C-reactive protein (CRP) Renal function (creatinine, urea) Liver panel (AST, ALT, ALP, GGT, bilirubin, total protein, albumin) All were assessed using automated analyzers. Fecal calprotectin was measured using a quantitative ELISA (cut-off < 50 μg/g) [14,16].
Serological tests	Viral serology included HBsAg Total anti-HBc Anti-HCV HIV-1/2 antigen/antibody (p24/Ab) Screening for latent tuberculosis was performed using an interferon-gamma release assay (IGRA, QuantiFERON test) [17,18].
Stool examinations	Stool was performed for *Salmonella* spp., *Shigella* spp., pathogenic *Escherichia coli*, and *Yersinia enterocolitica*. *Clostridioides difficile* toxins A and B were detected by enzyme immunoassay. Parasitological examination included three stool samples collected at 48 h intervals. Ziehl–Neelsen staining was performed to identify oocysts of *Cyclospora cayetanensis* under light microscopy [1,4,22].
Endoscopy	Gastroscopy was performed using standard high-definition video endoscopes. Colonoscopy and rectosigmoidoscopy were carried out using standard high-definition video endoscopes. Disease activity was graded using the Mayo endoscopic subscore (0–3), and disease extent was classified according to the Montreal classification [14,15,23].
Histopathology	Biopsies from the colon and duodenum were fixed in formalin, embedded in paraffin, and stained with hematoxylin and eosin (H&E). Colonic mucosa was evaluated for crypt architectural distortion, mucosal atrophy, and inflammatory infiltrates (plasma cells, eosinophils), in line with ECCO consensus criteria for ulcerative colitis [23,24]. Duodenal biopsies were assessed for intraepithelial lymphocytes and graded according to the Marsh classification.
Imaging	Contrast-enhanced computed tomography (CT) of the abdomen and pelvis was performed to assess colonic wall thickening, loss of haustration, and mesenteric lymph nodes. Abdominal ultrasound was used to evaluate bowel wall thickness and lymphadenopathy.

Abbreviations: CBC—complete blood count; CRP—C-reactive protein; AST—aspartate aminotransferase; ALT—alanine aminotransferase; ALP—alkaline phosphatase; GGT—gamma-glutamyltransferase; ELISA—enzyme-linked immunosorbent assay; HBsAg—hepatitis B surface antigen; anti-HCV—antibody to hepatitis C virus; HIV—human immunodeficiency virus; IGRA—interferon-gamma release assay; ECCO—European Crohn’s and Colitis Organisation; H&E—hematoxilin and eosin; CT—computed tomography.
